# Animal board invited review: advances in proteomics for animal and food sciences

**DOI:** 10.1017/S1751731114002602

**Published:** 2014-10-31

**Authors:** A. M. Almeida, A. Bassols, E. Bendixen, M. Bhide, F. Ceciliani, S. Cristobal, P. D. Eckersall, K. Hollung, F. Lisacek, G. Mazzucchelli, M. McLaughlin, I. Miller, J. E. Nally, J. Plowman, J. Renaut, P. Rodrigues, P. Roncada, J. Staric, R. Turk

**Affiliations:** 1 Instituto de Investigação Científica Tropical, CVZ – Centro de Veterinária e Zootecnia, Av. Univ. Técnica, 1300-477 Lisboa, Portugal; 2 CIISA – Centro Interdisciplinar de Investigação em Sanidade Animal, 1300-477 Lisboa, Portugal; 3 ITQB – Instituto de Tecnologia Química e Biológica da UNL, 2780-157 Oeiras, Portugal; 4 IBET – Instituto de Biologia Experimental e Tecnológica, 2780-157 Oeiras, Portugal; 5 Departament de Bioquímica i Biologia Molecular, Facultat de Veterinària, Universitat Autònoma de Barcelona,08193 Cerdanyola del Vallès, Spain; 6 Institute of Molecular Biology and Genetics, Aarhus University, 8000 Aarhus C, Denmark; 7 Laboratory of Biomedical Microbiology and Immunology, University of Veterinary Medicine and Pharmacy, Komenskeho-73 Kosice, Slovakia; 8 Department of Veterinary Science and Public Health, Università di Milano, Via Celoria 10, 20133 Milano, Italy; 9 Department of Clinical and Experimental Medicine, Division of Cell Biology, Faculty of Health Science, Linköping University, SE-581 85 Linköping, Sweden; 10 IKERBASQUE, Basque Foundation for Science, Department of Physiology, Faculty of Medicine and Dentistry, University of Basque Country,48940 Leioa, Bizkaia, Spain; 11 Institute of Biodiversity, Animal Health and Comparative Medicine, University of Glasgow, Garscube Estate, Glasgow G61 1QH, UK; 12 Nofima AS, PO Box 210, NO-1431 Aas, Norway; 13 Swiss Institute of Bioinformatics, CMU – Rue Michel-Servet 1, 1211 Geneva 4, Switzerland; 14 Mass Spectrometry Laboratory, GIGA-Research, Department of Chemistry, University of Liège, 4000 Liège, Belgium; 15 Division of Veterinary Bioscience, School of Veterinary Medicine, University of Glasgow, Garscube Estate, Glasgow G61 1QH, UK; 16 Institute of Medical Biochemistry, University of Veterinary Medicine, Veterinaerplatz 1, A-1210 Vienna, Austria; 17 National Animal Disease Center, Bacterial Diseases of Livestock Research Unit, Agricultural Research Service, United States Department of Agriculture, Ames, IA 50010, USA; 18 Food & Bio-Based Products, AgResearch, Lincoln Research Centre, Christchurch 8140, New Zealand; 19 Department of Environment and Agrobiotechnologies, Centre de Recherche Public – Gabriel Lippmann, 41 rue du Brill, L-4422 Belvaux, Luxembourg; 20 CCMAR – Centre of Marine Sciences of Algarve, University of Algarve, Campus de Gambelas, 8005-139 Faro, Portugal; 21 Department of Veterinary Science and Public Health, Istituto Sperimentale Italiano L. Spallanzani Milano, University of Milano, 20133 Milano, Italy; 22 Clinic for Ruminants with Ambulatory Clinic, Veterinary Faculty, University of Ljubljana, Gerbičeva 60, 1000 Ljubljana, Slovenia; 23 Department of Pathophysiology, Faculty of Veterinary Medicine, University of Zagreb, Heinzelova 55, 10000 Zagreb, Croatia

**Keywords:** Proteomics, farm animals, aquaculture, animal health, post-harvest

## Abstract

Animal production and health (APH) is an important sector in the world economy, representing a large proportion of the budget of all member states in the European Union and in other continents. APH is a highly competitive sector with a strong emphasis on innovation and, albeit with country to country variations, on scientific research. Proteomics (the study of all proteins present in a given tissue or fluid – i.e. the proteome) has an enormous potential when applied to APH. Nevertheless, for a variety of reasons and in contrast to disciplines such as plant sciences or human biomedicine, such potential is only now being tapped. To counter such limited usage, 6 years ago we created a consortium dedicated to the applications of Proteomics to APH, specifically in the form of a Cooperation in Science and Technology (COST) Action, termed FA1002 – Proteomics in Farm Animals: www.cost-faproteomics.org. In 4 years, the consortium quickly enlarged to a total of 31 countries in Europe, as well as Israel, Argentina, Australia and New Zealand. This article has a triple purpose. First, we aim to provide clear examples on the applications and benefits of the use of proteomics in all aspects related to APH. Second, we provide insights and possibilities on the new trends and objectives for APH proteomics applications and technologies for the years to come. Finally, we provide an overview and balance of the major activities and accomplishments of the COST Action on Farm Animal Proteomics. These include activities such as the organization of seminars, workshops and major scientific conferences, organization of summer schools, financing Short-Term Scientific Missions (STSMs) and the generation of scientific literature. Overall, the Action has attained all of the proposed objectives and has made considerable difference by putting proteomics on the global map for animal and veterinary researchers in general and by contributing significantly to reduce the East–West and North–South gaps existing in the European farm animal research. Future activities of significance in the field of scientific research, involving members of the action, as well as others, will likely be established in the future.

## Implications

Proteomics allows the study of proteins present in a given tissue or fluid (the proteome). It is of significant importance to numerous scientific areas, including animal and veterinary sciences. Despite this, proteomics has been limited in these disciplines due to a number of reasons, including cost, lack of good genomic data from many species of interest and a lack of awareness of the potential of this technology by animal scientists. Here we provide examples of successful applications of proteomics in animal production and health with insights into where farm animal proteomics-based research will be directed in the next few years, encapsulating contributions to this new technology through the existence of Cooperation in Science and Technology Action Proteomics in Farm Animals (www.cost-faproteomics.org).

## Introduction

Farm animal products, such as meat and milk and increasingly including products from the aquaculture industry, provide the basis for the source of protein in food for human consumption and contribute to a balanced diet for the majority of the population. With the importance of protein as the end product of animal farming systems, it is perhaps surprising that the use of the most advanced protein analytical technology, proteomics, has been relatively neglected in farm animal research (Eckersall and Whitfield, 2011; Eckersall *et al.*, 2012). This position has been addressed in the last 4 years by the creation of a European Cooperation in Science and Technology (COST) Action on Farm Animal Proteomics (FAP) www.cost-faproteomics.org, which has formed a network demonstrating the fundamental role that proteomics can and will have in farm animal and food research. This paper reviews the achievements of the COST action participants in relation to its three central and interconnected work group themes of (a) proteomics of animals and fish during production, (b) proteomics of the post-harvest changes as farm products are converted to food and (c) technological advances in proteomics and their potential for exploitation by the animal science community.

Proteomics can be used in virtually all areas of animal health, production and welfare assessment. This technology has (as demonstrated below) been used, for example, to characterize pathogen–host interaction in the disease of production animals (Martins *et al.*, 2012), assess the status of reproductive health (Souza *et al.*, 2012) and determine the dynamics of muscle growth (Doherty *et al.*, 2004). Similarly, in the assessment of post-harvest modification, proteomic investigations have been undertaken to assess alteration in fish muscle (Kjaersgard *et al.*, 2006) and in meat maturation, particularly in relation to product safety and verification of the species of origin (Paredi *et al.*, 2012). The utilization of the full range of applications of proteomic investigation is dependent on the necessary expertise being available, and a cohort of early-stage researchers in animal sciences has now been exposed to these advanced technologies. It is now important that their aspirations to apply acquired knowledge for the benefit of animal science research be recognized and facilitated.

**Figure 1:**
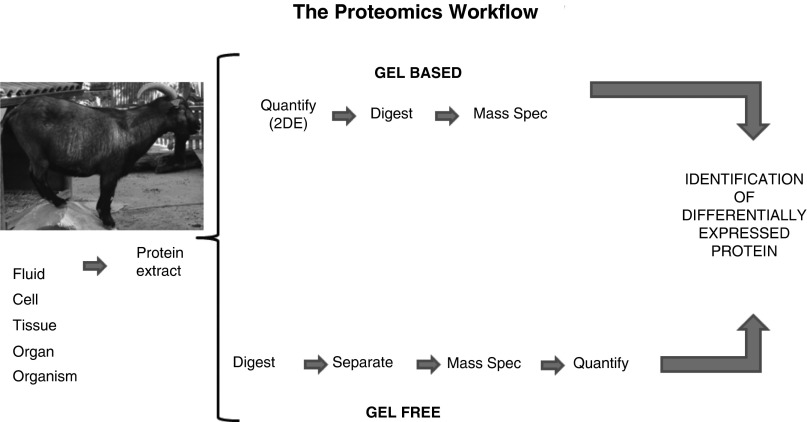
A schematic representation of the proteomics workflow. In proteomics, one of the two approaches are followed: gel based or gel free. In the first, individual protein expression is quantified using two-dimensional electrophoresis and individual proteins are digested with an enzyme, typically trypsin, and identified using MS. In the gel-free approach, the whole protein extracts are digested with trypsin, separated using chromatography and proteins of interest identified and quantified using high-throughput MS instruments. The latter approach is particularly suitable for species with reasonable coverage levels in databases (cattle, pig, sheep, chicken and salmon).

The current high potential for using proteomics in animal research has developed with advances in technology to separate and identify the proteins in a complex mixture such as it exists in biological samples. Separation of such a complex sample mixture can be performed either at the protein level (top-down approach) (Westermeier and Naven, 2002) or after digestion of the protein mixture to peptides (bottom-up approach) (Gevaert and Vandekerckhove, 2011), as summarized in Figure 1. For the first strategy, typically two-dimensional gel electrophoresis (2DE) is applied under reducing and denaturing conditions. This minimizes protein interaction and allows separation of proteins or protein subunits according to charge (isoelectric point) in the first dimension, followed by second-dimensional molecular sieving by molecular mass (in SDS-PAGE). After staining with visible/colorimetric or fluorescent dyes, protein spots are detected and evaluated in abundance by means of dedicated software. Alternatively, modern methods of pre-electrophoretic protein labelling with fluorescent dyes (fluorophores) allow direct detection of separated protein spots (Miller, 2012). Protein spots of interest, usually those that vary in intensity in a treatment- or disease-dependent comparison, are enzymatically digested into peptides. On the basis of their size and fragmentation pattern, subsequent MS analysis attributes them to particular proteins, aided by computer-based search in dedicated large databases.

In the second strategy, typically MS-based approaches are used in which proteins are digested into peptides, usually with trypsin whose cleavage sites are well known. Peptides are fractionated by chromatography, often in a multi-dimensional setup combining different separation principles (e.g. ion exchange-, reversed phase-, affinity columns). Quantitation in MS is based on isotopic or chemical labels that are introduced in a previous step into the organism, at the cellular level, by labelling the intact proteins or peptides, or performed in a label-free mode that requires high reproducibility of the analyses, besides the generally requested high mass accuracy and high-quality extensive databases (Nikolov *et al.*, 2012).

There are multiple varieties of gel-based and gel-free approaches, depending on the question under investigation, and they are often combined with sample prefractionation methods to reduce the complexity of the original sample mixtures (Posch, 2008; Miller, 2011). Studies have shown that both strategies provide complementary results (Anderson *et al.*, 2004). They also emphasized the importance of monitoring protein fine structure and modifications (e.g. glycosylation, phosphorylation), besides detecting protein concentration changes (Johnson and White, 2012; Ueda, 2013).

## Proteomics in farm animals during production in health and disease

### Proteomics in ruminants: health and disease

Proteomics encompasses new and emerging technologies that will facilitate sustainable animal production, quality and welfare. Although current trends are focussed on porcine and bovine species to identify biomarkers as predictors of food quality, animal stress and the detection and diagnosis of infectious diseases, recent activities are increasingly focussing on poultry and fisheries as well. Given the economic importance of dairy farming, the majority of proteomic studies in bovines have been carried out to elucidate pathogenic mechanisms of mastitis and endometritis. In addition, proteomics has been applied to identify markers of stress in order to monitor animal welfare in increasingly intensified farm management practices.

In fact, the primary goal in farm animal production is maintenance of animal health, fertility and growth in order to produce high-quality animal products such as meat, milk and eggs. Major changes in animal production over the years, such as housing conditions and the increase in the production of food of animal origin, have induced animals to respond to these demanding circumstances with a range of non-specific responses of the body, defined as stress responses (Blokhuis *et al.*, 1998). Stress in dairy cows is associated with increased susceptibility to infectious diseases such as mastitis, Johne’s disease, salmonellosis and bovine respiratory complex, as well as other production diseases including those affecting fertility, uterine diseases (metritis, retained placenta, prolapsed uterus), ketosis and milk fever, especially during the periparturient period (Nir, 2003). Most proteomic investigations of bovine mastitis, using strategies including 2DE followed by matrix-assisted laser desorption/ionization (MALDI) time-of-flight (TOF)/MS and liquid chromatography coupled with tandem mass spectrometry (LC-MS/MS), have been performed on bovine milk because of the relative ease of sample collection (Boehmer, 2011). These techniques have been used to evaluate the modification of milk proteins during mastitis in cows with naturally occurring infection (Hogarth *et al.*, 2004; Smolenski *et al.*, 2007) as well as in experimentally induced coliform mastitis (Boehmer *et al.*, 2008; Boehmer *et al.*, 2010a; Boehmer *et al.*, 2010b; Danielsen *et al.*, 2010). Hogarth *et al.* (2004) found significant increases in serum albumin and transferrin, concurrently with marked decreases in caseins, β-lactoglobulin and α-lactoglobulin, in the whey from cows with mastitis, suggesting that the transport of serum proteins into milk was because of the failure of the blood–milk barrier. Smolenski *et al.* (2007) identified apolipoprotein A-I (apo A-I), cathelicidin-I, heat shock 70kD protein and the acute-phase protein serum amyloid A (SAA) in milk fractions from cows with naturally occurring mastitis, indicating a local host response to infection in the mammary gland. Another acute-phase protein (APP), α-1-acid-glycoprotein, was identified for the first time by Boehmer *et al.* (2008) in normal and mastitis whey samples during a proteomic analysis investigating cows experimentally inoculated with *E. coli*. In a recent study, the serum proteome profile in cows with naturally occurring subclinical and clinical mastitis was investigated with three different yet complementary approaches in order to identify differentially expressed protein markers that are useful for early recognition of subclinical mastitis (Turk *et al.*, 2012). Results demonstrated differential protein expression in the serum of cows with both subclinical and clinical mastitis. These data indicate the involvement of the acute-phase response, oxidative stress, complement activation, protease inhibition and lipid metabolism by the innate immune system to combat infection by pathogens.

A number of these reports have described the proteome of milk whey after the removal of caseins, but other fractions of milk have also been the subject of proteomic analysis. Initial investigation into the milk fat globule membrane protein (Reinhardt and Lippolis, 2008) has been followed by analysis of milk exosome proteins (Reinhardt *et al*., 2012) and a combined investigation of the milk fat globule membrane, exosome and whey proteins in *S. aureus* mastitis (Reinhardt *et al.,* 2013). A total of 300 milk proteins were identified with links to host defence, with 94 being differentially regulated in mastitis.

### Pathogen proteomics

Proteomic technologies have also been used to provide novel approaches and insights into the pathogenic mechanisms of bacterial infection in farm animal diseases, which offer unique opportunities to study the proteome of bacterial pathogens during infection (Virgin, 2007). A limited number of proteomic studies have focussed on pathogen responses during clinical intramammary infections (Taverna *et al.*, 2007, Tedeschi *et al.*, 2009). Taverna *et al.* (2007) discovered major membrane-associated proteins in bovine mastitis *S. aureus* isolates that could be involved in the recognition of mammary epithelial cell receptors. Tedeschi *et al.* (2009) identified the three highly immunogenic proteins in bovine mastitis *S. aureus* isolates involved in virulence. Recent proteomic studies investigating different *S. aureus* strains isolated from cows with clinical and subclinical mastitis resulted in the identification of 15 proteins that exhibited variable expression in a range of *S. aureus* isolates (Wolf *et al.*, 2011).

2D electrophoresis was also applied to investigate the virulent state of *M. avium* subsp. paratuberculosis in which a direct comparison of the proteomes of *M*. *avium* subsp. paratuberculosis, scraped from the terminal ileum of ovine paratuberculosis cases, was made to the identical strain grown *in vitro*. This study identified a set of 10 proteins whose expressions are upregulated during natural infection (Hughes *et al.*, 2007), which may have implications for biomarker studies and therapy design strategies. The proteome of pathogenic leptospires, the causative agent of leptospirosis, expressed during urinary excretion from reservoir hosts of infection, is modulated to facilitate host evasion by diminution of antigen expression and increased expression of the virulence factor Loa22 (Nally *et al.*, 2007; Monahan *et al.*, 2008). Furthermore, proteomic technologies are compatible with novel extraction procedures to enrich for bacterial hydrophobic outer membrane proteins expressed during infection (Nally *et al.*, 2007; Crother and Nally, 2008). Finally, the continued development of novel proteomic approaches such as Capillary Electrophoresis-Mass Spectrometry (CE-MS) have the capability to identify panels of peptides that can be used for disease diagnosis and for differential diagnosis of the causative bacteria of the infections of the mammary gland (Mansor *et al.*, 2013; Albalat *et al.*, 2014). As with bovine species, a significant amount of proteomic studies have been performed on porcine species (de Almeida and Bendixen, 2012; Ceciliani *et al.*, 2014). In addition to its role during meat production, the porcine species is an important animal model for the study of disease in humans.

### Avian proteomics

An interesting field of application of proteomics is also the study of the pathogenesis of infectious disease affecting the avian species. The importance of this topic ranges from the economical aspect, to reduce the impact of avian diseases on production by characterizing the pathogenesis and by identifying new biomarkers of vaccines, to the need to study some avian diseases as a zoonosis, for example, avian flu, where human host adaptation signatures have been identified (Miotto *et al.*, 2010) and responses to the virus characterised in mice (Zhao *et al.*, 2012) and chicken (Sun *et al.*, 2014). Proteomics has been already utilized to study the pathogenesis of herpes viruses (Kunec, 2013), with a special focus on Marek disease (Thanthrige-Don *et al.*, 2010; Hu *et al.*, 2012), which is of particular interest as a model for human tumours (Buza and Burgess, 2007).

### Proteomics in aquaculture

Aquaculture has been ongoing for centuries, but this industry has undergone rapid and extensive expansion because of the rapid growth of the average seafood consumption per person in the last 50 years. To accommodate this demand, aquaculture companies are now breeding fish to improve traits such as their growth rate, conversion of feed into muscle, disease resistance, fertility and other features associated with food quality. Nevertheless, one of the main challenges faced by this industry is its impact on environmental sustainability where clearly a public intolerance to any potential new source of pollution or the further degradation of the natural environment may act as a drawback. Proteomics application in aquaculture is mainly focussed on nutrition, welfare and health management, as these have proven to be major constraints to an efficient production in aquaculture systems (Rodrigues *et al.*, 2012).

With regard to the nutrition source of farmed fish, there is a recent trend to move away from the traditional use of marine-harvested resources towards a diet-containing vegetable protein and oil sources. Although this reduces the impact on the marine-based food source, the growth rates and feed efficiency are compromised. However, proteomics is contributing greatly to a better understanding of the metabolic pathways affected by these dietary changes, as demonstrated in species like rainbow trout (Martin *et al.*, 2003; Vilhelmsson *et al.*, 2004; Keyvanshokooh and Tahmasebi-Kohyani, 2012), Atlantic Salmon (Sissener *et al.*, 2010; Morais *et al.*, 2012), Gilthead seabream (Ibarz *et al.*, 2010; Rufino-Palomares *et al.*, 2011; Siva *et al.*, 2012; Matos *et al.*, 2013) or *Diplodus sargus* (de Vareilles *et al.*, 2012). These studies were mainly focussed on fish liver and muscle with identified protein responses involved in glycolysis, amino-acid catabolism, energy and lipid metabolism, oxidative stress or the immune system.

Fish diseases are responsible for the main economic losses in aquaculture. These diseases are mainly caused by viral, parasitic and bacterial infections and significantly affect the production yield worldwide (Hill, 2005). Several pathogen detection methods (traditional, immunological, molecular, etc) have been extensively used, with vaccination being the main research area for disease prevention (Biering *et al.*, 2005, Hastein *et al.*, 2005, Sommerset *et al.*, 2005). Proteomics techniques have been assisting with this problem, especially at the level of development of new vaccines and disease diagnostics. Recent studies describe the isolation and the proteome analysis of the envelope proteins of the pathogen Iridovirus, which is responsible for the high mortality in cultured Grouper and also present in other Southeast Asian farmed species (Zhou *et al.*, 2011).

Proteomics is also an extremely valuable tool in assessing fish welfare through the development of new aquaculture practices that ensure that farmed marine animals can be reared in an environment that optimizes their capacity to cope with unavoidable challenges/stress, thus enhancing their state of welfare and health. The main target organ to be analysed is the liver, providing a window to their metabolic status, or body fluids like blood plasma that is easily retrievable from the live animal. Stress-related studies mostly focussed on the correlation between environmental sources of stress in aquaculture with proteome changes. They include high stock densities (Provan *et al.*, 2006; Alves *et al.*, 2010), handling (Alves *et al.*, 2010; Cordeiro *et al.*, 2012) and preslaughter stress (Morzel *et al.*, 2006). Studies focussed on the analysis of plasma proteins have concentrated on the detection and validation of welfare markers, with several proteins like microglobulins, macroglobulins, apolipoproteins, α1-antitrypsin, transferrin, plasminogen and complement system proteins among others being identified as possible candidates (Russell *et al.*, 2006; Brunt *et al.*, 2008; Bohne-Kjersem *et al.*, 2009; Kumar *et al.*, 2009).

### Parasite proteomics

The field of parasitology has been quick to exploit emerging proteomics technologies. The host–parasite interaction is particularly complex, being the result of two genetically distinct multicellular biological systems. The investigations have followed two directions. On one hand, proteomics focussed on the ‘parasite’, trying to identify the expression pattern of parasites, which is particularly challenging because of their different development stages. A second research direction followed the ‘host’ aspect, thus focussing on the complex dynamics that underlie the interaction between host immune system and parasite. Most, if not all, parasite antigens utilize the change of surface post-translational modifications (PTM) to modulate and possibly evade the host immune response. PTM are involved in many important molecular recognition processes including invasion, adhesion, differentiation and development (Frenal *et al.*, 2014). The advances in immunoproteomic techniques will provide new insights into the ‘host’ perspective of parasite infections, in particular for what concerns both innate immunity and biomarker discovery for diagnostic applications (Etebar *et al.*, 2013, Hasnain *et al.*, 2013).

### The metaproteome, an essential tool for the future of farm animal health proteomics

All those recent findings demonstrated the enormous impact of proteomics on the development of more accurate and cost-effective diagnosis and prognosis of animal farm diseases. There has already been movement from the single-parameter biomarker strategy to the proteomic-based multi-parameter diagnostic approaches (Campos *et al.*, 2012). However, metaproteomics, also known as the proteomics of the environment or of one particular community, is still an almost unexplored frontier to conquer in animal science. Metaproteomic analysis could provide identification and relative quantification of protein and protein families recovered directly from environmental samples or from a concrete ecosystem. This methodological approach aims to move from a functional understanding of a single species proteome elucidating the proteomic complexity from a larger community. Today, the latest MS technology in combination with sequencing capabilities and bioinformatic analysis has opened up new opportunities for translating metadata analysis into assessment of a health status (Erickson *et al.*, 2012). There are several advantages to consider: (i) metaproteomics could offer a phenotypic profile on samples preserving the meta-species environment; (ii) alteration in the microbial ecosystem (saliva, digestive tract, mammalian glands) in farm animals would not only provide functional information for diagnosis but also aid in evaluating stress responses; (iii) it could be of great interest to evaluate gastrointestinal function in ruminant and gastrointestinal disorders. The main disadvantage is that the developments in metaproteomics would still be linked to growth, achievements and methodological developments of metagenomics. In conclusion, this powerful new methodology would provide a deeper understanding of the farm animal microbiota in the context of functional host–microbe interactions and elucidate the molecular mechanisms linking the microbiome to host physiology. Proteomics and metaproteomic analysis are opening new opportunities for developing more robust and cost-effective diagnostics and prognostic methodologies.

## Proteomics in animal products post harvest

### Egg proteomics

Very limited proteomic research has so far been presented regarding quality control in chicken eggs. The literature is mainly focussed on ‘egg’ as a reproductive stage and not for its nutritional features. In particular, Qiu *et al.* (2012) investigated, through a 2DE-proteomic approach, the modification of egg proteins during storage. They described the differential proteome profile at three different storage temperatures (4°C, 20°C and 37°C) for 15 days. The most important result obtained was the degradation of albumin in relation to higher temperature, with the formation of a lysozyme–ovalbumin complex. Furthermore, the relative quantity of clusterin (apolipoprotein J) decreased with the same trend of increasing storage temperature, and it could, therefore, be used to assess egg quality. Another interesting paper (Rose-Martel *et al.*, 2012) applied LC-MS/MS proteomics to investigate the eggshell cuticle proteome, which represents the real barrier against the external environment and as a defence against mainly bacterial assault. This study of the protein composition of egg cuticle represents a milestone and highlights several parameters that can be used to assess egg quality. This is particularly important as even partial damage to the cuticle exposes eggs to microbial contamination from food-borne pathogens. Among the 47 proteins identified, two major proteins – namely, a Kunitz-like protease inhibitor and ovocalyxyn 32 – are known to present antimicrobial functions. These findings may be relevant for prediction/selection of eggs with increased resistance to food-borne pathogens. As well as the protein composition of the shells, egg white protein has been examined by proteomics. Wang *et al.* (2012) used a combined 2DE and LC/MS/MS proteomic approach to explore relative differences of egg white proteins across six different egg varieties. They found for the first time a quiescence precursor protein in eggs, previously identified only in chicken mesenchymal and fibroblast cells. These authors concluded that the proteome of different egg varieties has the same components; however, the relative abundance of individual proteins does vary between the different egg varieties.

### Milk

Several recent reviews have presented the application of proteomics in milk science, from description of a bovine PeptideAtlas (Bislev *et al.*, 2012a) to milk production, as well as on the quality and safety of milk (Roncada *et al.*, 2012). Some reports have also focussed their attention on the diagnostic value of milk proteomes in mastitis (Mansor *et al.*, 2013). More recently, Calvano *et al.* (2013) described a rapid and sensitive method to detect adulteration in milk, in particular to detect mixtures of powdered milk in liquid milk, both in raw and processed products. The same results can be obtained with 2DE-based proteomic analysis, but MALDI-TOF-TOF analysis is a reliable and fast method for this purpose. In particular, they identified diagnostic peptides of powdered milk with sensitivity of <1%. Nissen *et al.* (2013) described a powerful combined prefractionation method to characterize the bovine milk proteome. Authors were able to identify new proteins, and their data were supported by ELISA validation. The combination of accurate prefractionation methods, 2D-based proteomics, LC-MS/MS and ELISA can efficiently overcome the problems of measuring minor milk protein components, despite the large dynamic range of milk proteomes. Caira *et al.* (2013) reviewed and described different typical peptides useful to detect different types of milk and adulterations through the detection of *α*_S1_-CN variants. Furthermore, they set up a flow injection analysis electrospray ionisation quadrupole TOF analysis before dephosphorylation of casein that could also be used to assess different Mediterranean breeds. Negative energy balance (NEB) in cattle directly influences milk composition, with respect to both proteins and lipids. Lu *et al.* (2013) applied a proteomic and metabolomic approach to study NEB in milk. These authors showed that milk from these cows has an increased quantity of acute-phase proteins, galactose–1–phosphate and unsaturated fatty acid, in comparison with milk from cattle with a good energy balance. These observations have provided some insight into the potential mechanisms involving stomatin and galactose-1-phosphate in NEB dairy cow’s milk and demonstrate a relationship between metabolism and the quality of milk.

Although safety of dairy products is a prerequisite for the industry, food-borne diseases continue to be one of the most important causes of disease and fatalities in humans (Bassols *et al.*, 2014). Proteomics can help improve the detection of pathogens in food samples – for example, MALDI-TOF MS for diagnostic microbiology is a successful example of how proteomics can win this global challenge. Geng *et al.* (2006) presented an interesting paper about the detection of one of the more dangerous food-borne pathogens, *Listeria monocytogenes*, directly on selective-enrichment broth. MALDI TOF MS was successfully applied to detect pathogens in blood, but the real challenge was to apply these methods in complex pathogen systems as in food matrices using the SARAMIS database, with an algorithm optimized for the rapid detection of Listeria-contaminated food matrices.

### Cheese

In dairy products, especially for cheese, there has been a rapid increase in research using proteomics to study processes such as cheese maturation in order to improve the quality of production. For example, Hinz *et al.* (2012) used a 2DE-based proteomic approach to correlate the differences in the proteolysis of milk proteins during lactation stages. The authors correlated the proteomic pattern of the relative production of cheddar cheese derived from different time points of the lactation stages. Interestingly, they identified some proteins that could be useful to assess seasonal quality. Wedholm *et al.* (2008) described protein markers of the cheese yield through proteomic analysis. In particular, they highlighted several proteins related to the production as a specific *β*-CN fragment, an isoform B of *β* lactoglobulin, and other whey proteins as lactoferrin and vitamin D-binding proteins. However, the roles of these proteins were not completely explained. Regarding the role of casein fragments in poorly coagulating milk for cheese production, another interesting proteomic work has been published (Jensen *et al.*, 2012). Reduced levels of phosphorylation of *α*_S1_-CN form (*α*_S1_-CN 8P) are related to poorly coagulating and non-coagulating milk, together with decreased levels of glycosylated *κ*-CN forms.

### Meat: poultry

Although regarded as a major food source for humans, proteomics in poultry and fish science is still lagging behind that for other livestock species. Proteomics in poultry has focussed on basically two fields of research, meat quality and the study of infectious diseases. Differences in raw and cooked poultry meats were determined by means of proteomics (Montowska and Pospiech, 2013), which also provided the tools to identify new protein markers associated with slow and fast growth rates of a different genetic line (Phongpa-Ngan *et al.*, 2011). Proteomics was also useful to unravel the molecular basis of some protected designation of origin meat products, such as French *foie gras* or local chicken breeds (Zanetti *et al.*, 2010 and 2011), paving the way for future application of proteomic techniques in food quality certification (Theron *et al.*, 2011). The derangement of *postmortem* alteration such as those that drive to pale soft exudative meats can also be analysed by proteomics (Molette *et al.*, 2003). Given the background of the growing sensitivity of consumers and of policy makers on animal welfare, future applications of proteomics for the identification of biomarkers of stress and welfare after transport, for example, or preslaughtering procedures, are also envisaged (Hazard *et al.*, 2011).

### Meat: ruminants and pigs

The protein components in meat, meat-derived products and fish are the most important determining factors for the eating quality, although other components also add to the positive (or negative) effects of the food product. Although total protein amount is an important attribute of meat, the composition of proteins is even more important for the eating quality. This explains why proteomics has become increasingly used as a tool to describe, understand and improve protein-based foods during the last decade. This is also reflected by the increasing number of scientific review papers appearing in the field (Bendixen *et al.*, 2005; Hollung *et al.*, 2007; de Almeida and Bendixen, 2012; Paredi *et al.*, 2013). The composition of proteins is partially determined by the genetics of the animals. It has been known for decades that specific genes influence meat quality, such as the calpain genes that have a major impact on meat tenderness, as recently reviewed (Warner *et al.*, 2010). However, it is also well known that eating quality is strongly influenced by other *ante*- and *postmortem* factors. To understand product quality, it is essential to understand the molecular mechanisms involved and fundamentally to obtain knowledge about the protein composition. The conversion of muscle to meat is a complex sequence of events causing a gradual change spanning hours and days *postmortem*, depending on muscle type and species. For processed meat and meat products, other factors will also have an impact. Understanding how different factors influence the quality of the product will aid in the development of new procedures and practices for driving the *postmortem* development or product processing in the desired direction. There are several recent examples where proteomics has been used as a tool to explain food quality variation.

### Comparison of breeds with different production properties

A number of studies using proteomic tools have been undertaken to analyse why different pig breeds are associated with extensive differences in meat quality (reviewed in de Almeida and Bendixen, 2012). One example is the comparison of the native Italian Casertana pig breed with the Large White breed using both proteomics and metabolomics (Murgiano *et al.*, 2010; D’Alessandro *et al.*, 2011; Marrocco *et al.*, 2013). The Casertana breeds grow slower and have more backfat and intramuscular fat than do fast growing and leaner breeds like the Large White (Zullo *et al.*, 2003). The slower *postmortem* pH decline in meat from Casertana pigs and differences in metabolic rate were supported by the differences in protein abundances and levels of specific metabolites. Higher levels of glycolytic enzymes and increased lactate accumulation were observed in the Casertana breed. On the other hand, meat from the Large White had higher expression of genes involved in cell cycle and muscle growth, also supported by proteomics. Furthermore, the metabolomics studies revealed an increase in Glutathione and Glutathione disulphide in Large White compared with Casertana pigs.

### Molecular changes during *postmortem* storage

Conversion of muscle to meat starts immediately after slaughter of the animal. Following slaughter, the muscle will use the remaining energy stored and the muscle proceeds into *rigor mortis* before proteolytic enzymes start to degrade the myofibres (reviewed in Huff Lonergan *et al.*, 2010). Another important event is the onset of apoptosis (Ouali *et al.*, 2006; Kemp *et al.*, 2010; Hollung *et al.*, 2014). All these events depend on external and internal factors and the rate of the specific events is both muscle and species specific. *Postmortem* changes in the porcine muscle proteome include both metabolic enzymes and myofibrillar proteins (Lametsch and Bendixen, 2001; Lametsch *et al.*, 2003; Morzel *et al.*, 2004). In addition, similar studies performed in beef show that many metabolic enzymes, cellular defence and stress proteins change in abundance *postmortem* (Jia *et al.*, 2007; Laville *et al.*, 2009). The proteome analyses support a shift in energy metabolism *postmortem*. Aerobic metabolism continues for a short period after slaughter in the muscle, demonstrated by increased abundance of enzymes involved in the glycolytic pathway. The resulting production of lactate and protons contributes to the pH decline. Several studies have shown that the abundance of heat shock proteins (HSP) 27 and 70, both known inhibitors of apoptosis, is decreased (Picard *et al.*, 2010; Guillemin *et al.*, 2011b), eventually increasing apoptosis in *postmortem* muscle. For beef, tenderness is the most important eating quality parameter determined by consumers (Miller *et al.*, 1995). The search for reliable markers of tenderness in beef using various proteomic approaches has been a major focus for several research groups over the last decade (Bjarnadottir *et al.*, 2012; D’Alessandro *et al.*, 2012a and 2012b; Sierra *et al.*, 2012). Several markers have been proposed, and these are involved in different biological pathways or functions such as myofibril structure, proteolysis, oxidative stress resistance, apoptosis or energy metabolism. Proteins belonging to the HSP27 and HSP70 families are among the most promising tenderness markers so far (Guillemin *et al.*, 2011a), although these proteins are involved in a number of different cellular responses. In addition to their anti-apoptotic role, these proteins are protectors of myofibrillar proteins like desmin, actin, myosin and titin.

### Proteomics of processed meat

Production of cooked ham involves several processing steps such as injection of brine with different salt content, and tumbling of the muscle, before the final cooking step. Pioselli *et al.* (2011) observed significant differences in myofibrillar muscle protein composition of the exudates when using different salt concentrations and tumbling times. An initial salting step is also a central part of the processing when producing dry-cured hams. Proteins are released in the exudate formed in the initial salting step of Italian Parma ham production, and the processing conditions influence significantly the release of myofibrillar proteins in the exudate (Paredi *et al.*, 2013). Skrlep *et al.* (2011) found a correlation between salt content and 45 proteins in the *biceps femoris* muscle of dry-cured ham. Most of these proteins belong to the myofibrillar protein fraction. Release of amino acids and peptides by proteolysis of myofibrillar proteins is important for taste and odour in dry-cured hams. Several peptides derived from actin, myosin light chain and creatine kinase are among the peptides released during ripening of Spanish dry-cured hams (Sentandreu *et al.*, 2007; Mora *et al.*, 2009a and 2009b; Escudero *et al.*, 2013).

### Eating quality of fish

The eating quality of fish depends highly on the textural properties of the fillet, such as firmness flakiness, juiciness, oiliness and fibrousness. So far, only a few studies addressing the eating quality of fish have been based on proteome studies. Rodrigues *et al.* (2012) reviewed this subject extensively, with Jessen *et al.* (2013) identifying several proteins in rainbow trout muscle that are correlated to the textural attributes.

## Current and future technical advances for animal proteomics

The evolution of proteomics this last decade is strongly correlated to both technology and bioinformatics advances. At present, proteomic studies allow the high-throughput analysis of thousands of proteins leading to a huge amount of data (Steen and Mann, 2004; Schulze and Usadel, 2010; Zhang *et al.*, 2013). The rise of data-independent techniques on increasingly sensitive MS reinforces this trend. MS data need to be processed by adequate bioinformatic tools in order to extract biologically relevant information. When applied under ‘good practice’ and following the up-to-date guidelines, detection, identification and quantification of proteins can be achieved with precision and reproducibility (Martinez-Bartolome *et al.*, 2013). A range of techniques and workflows are readily applicable to FAP (Eckersall *et al.,* 2012).

Furthermore, data interpretation relies on knowledge of proteins that is tightly related to genomics, transcriptomics and metabolomics data. It is now relatively straightforward to apply tools developed for human and mouse genomes to a few well-assembled and annotated livestock genomes. However, there are several unsolved issues in making these genome resources relevant to agriculture. One important challenge is the management and representation of thousands of variant genomes per species. Existing efforts to adapt bioinformatics to the needs of different applications of biotechnology have remained dispersed, missing the interoperability and integration. These application criteria characterize resources for major biomedically relevant organisms and lack attention to the usability needs that correspond to different stakeholders such as breeders, biotechnology small and medium-sized enterprises, food industry and environmental or marine biology monitors. Protein functional annotation and omics data integration still need rigorous exploration and streamlining.

### Sample preparation considerations

Sample preparation is a critical step in any proteomic approach where limitations are generally sample-type dependent more than species specific. Owing to the broad spectrum of animal and veterinary sciences, including physiology, productive aspects and disease/parasite tolerance, specific methodologies have been or need to be developed. Many suitable protocols for a wide variety of species and tissues are available from the papers covered by this review. In addition, methodologies from human proteomic studies are often directly applicable to animal proteomics in the majority of circumstances, and general considerations of sample preparation have been reviewed (Finoulst *et al.*, 2011; Bantscheff *et al.*, 2012). As it is not possible here to review all the possible protocol variations, we want to highlight, in this section, the common key steps that need to be applied and controlled before proteomic analysis is undertaken and identify the limitations that can arise when dealing with such different starting materials. Soares et al. (2012) have recently reviewed the protein identification strategies and particularities of farm animal species.

The reproducibility of results and therefore the number of replicates in a study is of major concern in proteomics as in all biological studies. Replicates should be sufficient to allow for both biological and technical variability. Statistical approaches have been described that take into account small sample numbers, which may be necessary in studies on farm animals (Schwammle *et al.*, 2013). The amount of starting material and its heterogeneity need to be carefully evaluated according to the source of sample. For cell-culture experimentation, mean protein content per cell and number of cells have to be known, as well as protein concentration for biofluids. For tissue/biopsy analysis, the homogeneity of the samples is crucial and has to be comparable. An ideal scenario would be when proteomic investigation follows microdissection from histological sections, thus ensuring the tissue and cellular source of the sample. Total or selective protein extraction and purification should not introduce protein modifications, and endogenous enzymatic activities need to be prevented by adding inhibitors. Classic biochemical techniques combined with protein precipitation are widely used for sample preparation. Maximal protein solubilization and denaturation need to be achieved before applying chemical modifications to the proteins being analysed (disulphide bridges reduction/alkylation, labelling experiment, enzymatic digestion before LC-MS analysis). Exogenous proteins, labelled analogues or housekeeping proteins should be used as internal standards to assess methodological repeatability and reproducibility (Domon and Aebersold, 2010; Meng and Veenstra, 2011; Maiolica *et al.*, 2012; Picotti and Aebersold, 2012).

At the analytical point of view, when workflow uses entire protein separation (1D and 2DE), protocols often need to be adapted, as entire proteins can have very different physico-chemical properties (isoelectric point, hydrophobicity, molecular weight, tendency to aggregate). Gel-free techniques are generally more robust because they are completely automatized and directly applicable to a wide range of studies, as the enzymatic digestion of proteins into peptides leads to a more homogeneous set of molecules sharing similar properties. For both gel-based and gel-free approaches, the main analytical issues are the protein expression dynamics and complexity. When several enrichment methods help analyse targeted groups of proteins, the sample complexity is more dependent on the analytical power separation of the workflow used. Multidimensional separation techniques (2DE and 2D LC) coupled directly or not with MS can today identify and quantify about 4000 proteins (false discovery rate, 1%) per analysis hour covering 4 to 6 orders of protein abundance (Hebert *et al.*, 2014). The new-generation MS can further increase the separation power by introducing additional separation in gas phase (IMS) or by dramatically increasing the speed acquisition, mass accuracy and mass resolution.

### Differential proteomics approaches: gel-based proteomics

Although an abundance of proteomic data arise from gel-free approaches, the gel-based method can still provide important information on the status of the protein itself. As stated by F. Lottspeich, ‘many peptides are identically found in functionally completely different proteins’ (Lottspeich, 2009) – that is, a processed protein (cleaved protein, for example) is easily seen on a gel, whereas in the gel-free approach cleaved and non-cleaved proteins will give the same peptide pattern. At present, the most recent technical advance in the gel-based method is the difference gel electrophoresis or difference in gel electrophoresis, allowing the separation of two to three samples in a same gel and/or the use of an internal standard, thus improving the statistical reliability and decreasing the hurdle of reproducibility between gels. Excluding the use of litres of buffer (e.g. in the horizontal electrophoretic separation) and enabling the use of precast gels have been regarded as major advancements in the handling of gels. Besides the classical approach aiming at identifying proteins showing different levels of abundance when comparing multiple conditions, gels can be used for different aspects. This includes the detection of PTMs using specific staining and/or labelling procedures (Cerny *et al.*, 2013), estimation of native protein activity, separation of native proteins, separation of complexes or supercomplexes from soluble or membrane proteins (Wittig and Schaegger, 2008) and validation of results using antibodies.

### Shotgun LC-MS

The shotgun approach aims at comparing complete proteomes in terms of protein abundances between different conditions without any *a priori* use of an LC-MS workflow. This analysis often called the discovery approach highlights a set of biomarkers that needs to be further validated by alternative methods. Label-based or label-free methods can be applied. When introduced early in the sample preparation, label-based methods are preferred for analyses that require extensive prefractionation/enrichment approaches, as lack of reproducibility of the above methods can be compensated for by a unique processing of the mixed sample. However, only a limited number of conditions can be analysed with such techniques. On the contrary, label-free methods are generally applied for large studies with a high number of samples to be analysed. With this approach, one should ensure that the whole analytical procedure is standardised and uses sufficient quality control standards for all critical steps (protein extraction/purification, protein digestion, LC-MS analysis) (Martinez-Bartolome *et al.*, 2013). At the MS acquisition point of view, two main approaches exist. In one, the data-dependent acquisition implies an isolation in gas phase of each ion (peptide) to be further fragmented in tandem MS to collect peptide sequence information. The MS/MS speed acquisition directly limits the power of this approach, as just a part of the detected MS signals are selected for MS/MS sequencing. In addition, the lack of reproducibility of the selected ions for MS/MS acquisition induces loss of information when comparing several analyses. In another recent approach, data-independent acquisition was introduced to overcome these limitations. This acquisition mode is a parallel process where all MS signals are further submitted to MS/MS fragmentation without any precursor selection. This method has the advantage of acquiring all the information, therefore leading to higher acquisition reproducibility and the possibility of processing data completely and independently without having acquisition-dependent bias.

### Targeted proteomics in farm animal research

Although shotgun-based approaches in proteome research have been the gold standard for biomarker studies in the past decade, it has become increasingly clear that this approach is not very efficient for the quantitative analysis of low abundant proteins within complex biological samples (Malmstroem *et al.*, 2007). This means that many proteins of immediate relevance to research and surveillance of farm animal health, such as cytokines and their receptors, have hardly ever been observed by shotgun proteomic analyses of samples such as bovine milk (Danielsen, *et al.*, 2010; Boehmer, 2011). Moreover, because of the high cost and extensive instrument time required, typically only a relatively small number of biological replicates are analysed in global proteome experiments. Hence, much current focus has been on developing more selective and accurate methods for validating biomarkers and especially those that are present at low concentrations in samples. Furthermore, validation of methods for absolute quantification is essential for clinical as well as research applications in veterinary medicine or animal health studies. Absolute quantification is also necessary for correlating protein expression data across different biological samples, as well as across multiple experiments, instruments and laboratories, thus supporting more quantitative and hypothesis-driven proteome research.

Selected reaction monitoring (SRM) and the related multiple reaction monitoring are currently the favoured methods for biomarker verification and for targeted and absolute quantification approaches. The SRM method is essentially based on tandem MS analyses, where only a few selected peptides (typically 2 to 4 peptides/protein), which are unique to the protein of interest, are measured. Typically, triple quadruple instruments are used because of their high selectivity and sensitivity of both parent and fragment ions. By scheduling the MS methods, improved sensitivity is achieved by analysing specific peptides only within their optimal retention time windows. Depending on the sensitivity and accuracy required, >100 peptides, signifying 30 to 50 specific proteins, can be targeted and measured in one single LC-SRM/MS analysis of a complex sample, loaded over a 30 to 60 min LC gradient online to the MS (Pan *et al.*, 2009). Moreover, SRM methods cover a much wider dynamic range than shotgun-based MS (Kirkpatrick *et al.*, 2005), and an added advantage is that absolute protein quantities can be measured by spiking in known amounts of heavy-labelled peptide analogues, whereby the absolute amounts of the targeted peptides in the biological samples can be calculated.

SRM approaches have been proven to be highly successful for studies of specific proteins in complex biological matrices such as plasma, tissue samples and yeast down to sub-femtomolar levels (Huttenhain *et al.*, 2012; Picotti and Aebersold, 2012). SRM therefore provides a complementary and sometimes superior alternative approach to ELISA for biomarker validation, because of its high selectivity and specificity, low cost of reagents and the excellent capability of performing multiple assays in a single measurement (Pan *et al.*, 2009). So far, only very few examples of the use of SRM methods in farm animal sciences have been presented. These have been limited to bovine studies, such as the analysis of milk samples, where an SRM approach was used to specifically quantify membrane proteins in the milk fat globule (Affolter *et al.*, 2010), and a method paper that presented the quantotypic properties of a panel of known and potential bovine host response proteins studied in bovine udder samples from healthy as well as lipopolysaccharide-challenged cows (Bislev *et al.*, 2012b). Ongoing work at Aarhus University currently includes developing panels of SRM assays aimed for veterinary diagnostic purposes. These initiatives cover work on equine, bovine and porcine proteins with immediate relevance to farm animal research and clinical diagnostic purposes.

### Resources for SRM assay development

The PeptideAtlas repository (www.peptideatlas.org) currently provides the largest open-source compendium of observed peptides and can be easily browsed for proteotypic peptides suited for SRM-based designs. Although human, yeast, drosophila and honey bee proteomes are among the best covered species, pig, cow and horse proteomes are also relatively well represented in this resource, which greatly facilitates peptide selection for SRM-based method development. For pig, the PeptideAtlas currently covers >8000 proteins and 50 000 distinct peptides, from >20 different tissues. The bovine PeptideAtlas mainly represents milk and mammary gland proteomes, but includes also the proteomes of immune cells, as well as of inflamed claw and joints tissues. The bovine PeptideAtlas comprises 1921 proteins and 8559 distinct peptides (Bislev *et al.*, 2012a). The equine PeptideAtlas covers >2600 proteins and 24 000 peptides, collected from >25 types of tissues and body fluids (Bundgaard *et al.*, 2014). These resources currently provide a valuable tool for the selection of proteotypic peptides suitable for quantitative studies.

The SRM atlas (http://www.srmatlas.org) comprises an open-source compendium of validated SRM peptide assays and provides an invaluable resource for implementing new SRM-based methods for biomarker validation. For yeast, human and mouse proteins, a near to complete proteome coverage is presented, in the sense that >95% of all known proteins are represented by at least one unique peptide in these SRM assay repositories. On the other hand, for farm animal proteomes, no entries have yet been made, and unfortunately progress in this field is greatly hampered by the lack of triple quadrapole instrumentation and by the relatively few laboratories that work with FAP, compared with the domain focussed on research on human samples. Building these SRM resources for farm animals is even more important in the light that for veterinary species, for example horse, there is a severe lack of adequate and robust antibody-based methods for monitoring even the most important and relatively high-abundant diagnostic protein markers such as acute-phase proteins (Kjelgaard-Hansen and Jacobsen, 2011). Thus, the SRM-based methods may provide significant progress in veterinary diagnostics in the near future.

### Quantification concatemers (QconCAT): an interesting approach for large-scale studies

Because synthetic standard peptides are expensive and therefore a main scale-limiting factor, alternatives to commercially available peptides have been presented. An interesting approach is represented by the expression of QconCATs of tryptic standard peptides (Beynon *et al.*, 2005), which has proven useful and highly cost efficient when many biological replicates are to be studied. In this strategy, a chimeric protein is designed as a concatamer of tryptic standard peptides, which are metabolically labelled in *Escherichia coli* using stable isotopes. ^13^C-labelled Arg and Lys are used, which adds a mass difference of +6 Da to each peptide mass spiked into biological samples, to provide absolute quantities of the analysed peptides. As all isotope-labelled peptides are present in a single copy within the chimeric protein, they will inherently be present at the exact equimolar quantities within the analyte sample.

This QconCAT-based SRM approach was successfully used to study a panel of 20 bovine host response proteins, which may be relevant as early indicators for bovine mastitis (Bislev *et al.*, 2012b). The method proved successful for efficient and absolute quantification of 17 of the targeted proteins within healthy and inflamed mammary gland tissues, and provides a method that supports multiplexed and antibody-independent absolute quantification of inflammation-related proteins in the cow. This method suggests a promising approach for studies of large cohorts of animals with naturally occurring mastitis infection, and indeed for more thorough investigation of biological variation in these markers that are potentially relevant for veterinary diagnostics. Ongoing work is currently implementing this approach to monitor the time-course of individual animal response to naturally occurring mastitis events.

## The future of proteomics in animal science

As can be seen from the preceding reviews of the applications and technological developments that have an impact on the use of proteomics in animal science, there have been major developments over the last few years. This has coincided with a COST Action on FAP, which has nourished the development of the use of proteomics technology and a realization of the potential uses in animal research. Proteomics is closely linked, but it is a distinctly different analytical approach from other developing ‘omic’ sciences, such as genomics, metabolomics and transcriptomics, and together with the bioinformatics required to integrate the data output of analysis is integral to a systems biology approach. Each of these ‘omics’ requires development of a different skill set and, even though there is valuable interchange between these disciplines, it is important that expertise is established in all so that applications and technical standards can develop. Although there have been tremendous strides in the applications of genomics and transcriptomics in farm animals, it is important that proteomics takes its rightful place alongside these technologies.

A major stimulus has been delivered to the use of proteomics in animals by the recent activity of the COST Action on FAP (www.cost-faproteomics.org) in establishing an international forum for technology development. The accomplishments of this group have largely contributed to the above review, and by linking centres of proteomic excellence with active researchers in farm animal science many areas have been advanced. Current achievements include proteome maps established for plasma and tissue in a variety of production animal species, such as cattle and swine. Proteomic investigations have also been undertaken to assess meat maturation and to monitor post-catch changes in the protein profile of fish muscle. Proteins in milk have been closely examined by proteomics as it is important to determine changes that occur in its protein composition for the assessment of health status, quality and safety of dairy products. Proteomics has been used to characterize disease states in production animals and to determine the origin and source of feed products. Proteomic strategies have been used to measure the dynamics of muscle growth in poultry and advance farm animal reproduction. Proteomics has been used to monitor change in muscle protein as it matures to meat, with particular reference to the changes that take place in the meat proteome in the speciality smoke-dried meats of Southern Europe. Proteomic technologies have been adopted to monitor food composition, authenticity and safety – for instance, by identifying accurately the species of meat in processed food to enforce accurate food labelling and prevent false labelling of meat products as in recent cases where horse meat was sold as beef.

The COST action was established to overcome the principle hurdles to the applications of proteomics in farm animal science, which were that there were no clear roles for this approach in animal science and that accessing the technology was beyond the reach of animal science groups. The former has now been clearly removed with the extent of the potential of proteomics being clearly seen by the success of dedicated conferences and publication of a growing literature and successive reviews. The latter problem in access to the technology has been addressed, and, although there are perhaps still too few centres of proteomic excellence devoted only to animal science research, there is now a network of laboratories, with the most advanced equipment, which has shown the benefit of collaboration with the animal science community.

### FAP COST action activities

A notable success of the Action has been Short-Term Scientific Missions (STSMs) in which early-stage researchers have been able to expand their horizons by travelling to the leading proteomic laboratories in other countries to learn technology and undertake collaborative research. Over 50 missions have taken place with a great diversity of research applications with respect to farm animals using proteomic technologies in the wide range of funded STSMs in each of three different working group packages. Examples of funded STSMs focussing on animal health include the following: the characterization of circulating in comparison to pulmonary serum amyloid A protein in pigs with respiratory disease; proteomic analysis of serum from cattle infected with *Mycobacterium avium* subspecies paratuberculosis; analysis of biomarkers of disease in gilthead seabream; proteomic analysis of young in comparison to old osteoarthritic equine cartilage; and proteomic analysis of a range of pathogens, including zoonotics, to elucidate the pathogenic mechanisms of infection. Examples of funded STSMs focussing on food production, quality and food safety include the following: the impact of diets on adipose tissue in goats; proteomics of the blue mussel *Mytilus edulis* affected by water pollutants; metaproteomics of cheese and dry-cured ham; and the identification of fish proteins acting as allergens in humans. Examples of funded missions focussing on the technical aspects of animal proteomics include the following: training in the use of bioinformatic tools; analysis of data using different databases; quantitative proteomics; molecular modelling; and development of new algorithms for data analysis.

In addition to the STSMs, early-stage researchers in animal sciences have been introduced to the concepts of proteomics by attending training schools. These have been organized to illustrate the applications of proteomics to animal science and pathology. The core of the courses addressed to the application of MS for protein identification after on-gel and off-gel techniques constitutes the following: 2DE gel analysis, digestion of spots and MS analysis; introduction to MALDI, sample preparation, identification and presentation of results; and Orbitrap technology, which offers increased analytical performances for the analysis of peptides and proteins. Further schools have focussed on bioinformatics in order to enhance full interpretation of the output from separation and MS analysis. The most popular training schools in the COST action have been in the basic and advanced uses of bioinformatics covering MS data management and running of search engines; data format and existing toolboxes for converting, merging and preprocessing data from several sources (LC/MS-MS, iTraq, MALDI, etc.); the use of biological databases, such as UniProtKB, PeptideAtlas, Pfam and STRING; the creation of Perl or Python scripts; and an overview of protein–protein interaction programmes. Overcoming the limited nature of genomes of species such as seabream and Atlantic salmon is one pivotal problem encountered in the interpretation of MS data for animal species. Indeed, ensuring that the bioinformatic tools are as advanced as possible for the analysis of protein and peptides from livestock and wild and farmed fish species would be a valuable asset in the development of proteomics for farm animals. It is also important that, as investigations in animal species develop, the repositories of data such as proteome maps and peptide atlases are collated, curated and integrated with ontology databases. Indeed, the Bioinformatics for FAP highlighted two important problems for the discipline. First, the increasing importance of studies on PTM of proteins and, second, the need to create specialized spectral libraries that would be the key to designing sensitive MS-based assays. The success of the Skyline software (https://skyline.gs.washington.edu/labkey/project/home/begin.view) in proteomics emphasizes this trend and can be used for animal proteomics analysis.

In addition with Work Group meetings on animal health in production, post-harvest modification of muscle and meat, Training Schools and Workshops, the Action has developed a cohort of expertise in proteomics, which means that there is a generation of researchers who are able to coherently apply their working knowledge of proteomics to scientific investigation into animal health and production science.

A specific use for proteomics in the identification, development and validation of biomarkers of disease (or physiological change) has been highlighted by interaction with an International Meeting of Animal Clinical Pathologists (ISACP and ESVCP, Ljubljana, Slovenia) and General Proteomics (Seprot, Barcelona, Spain). The former groups comprise the laboratory professionals responsible for animal health testing and who expressed particular interest in the rapid development of proteomics for biomarker discovery as illustrated by studies of milk proteome in cows with mastitis and blood plasma proteome in disease and health (Hogarth *et al.*, 2004). The huge problem of NEB and fatty liver in cattle is addressed by a proteomic study of liver samples demonstrating downregulation of enzymes of *β* oxidation in cows with fatty liver, which are also significantly more exposed to oxidative stress (Kuhla *et al.*, 2009). Finally, proteomics techniques and strategies dealing with complex samples were introduced to clinical pathologists indicating the important problems encountered when trying to determine low-abundance proteins in serum (Marco-Ramell and Bassols, 2010).

### International collaborations

It is of critical importance and should be clearly demonstrated that proteomic analysis is not only the preserve of the more advanced laboratories in the West of Europe. The Action has therefore helped to restrain the worrying brain drain from Eastern Europe and reverse poor participation of scientists from the region in the EU’s research projects. For example, the 3rd FAP Annual Conference in Kosice, Slovakia, reversed the tradition of the east to west travel while disseminating advanced concepts of proteomics and bioinformatics. Through this and other FAP meetings and workshops held in Ljubljana, Slovenia, and Istanbul, Turkey, a growing interest has developed between colleagues keen to collaborate and travel to implement the application of the best analytical science to advancing animal science.

Although there has been more recent stimulus to the development of proteomics of animals in Europe, there have been valuable links with other continents and the science is evolving across the globe – for instance, in the Americas (Burgess, 2004; Lippolis and Reinhardt, 2008), Asia (Kim *et al.*, 2007; Lu *et al.*, 2012) and New Zealand (Plowman *et al.*, 2007; Smolenski *et al.*, 2007). One model example is the cooperative link established with New Zealand. For historical reasons, New Zealand, and particularly AgResearch, the New Zealand partner of FA1002, has had a strong tradition in wool proteomics, a difficult sample that involves the access to dedicated protein databases and challenged by the correct identification of proteins that, albeit their limited number, are extremely similar. Such expertise is matched by very few other laboratories worldwide. This action made possible one Reciprocal STSM (basically an STSM involving a European researcher who conducted a short stay in one of the reciprocal agreement COST countries: Australia, South Africa, Argentina and, in this particular case, New Zealand). The application process was straightforward, and the process from application to decision stage concluded very quickly. This brought together a Portuguese researcher’s interest in seasonal weight loss with AgResearch expertise in wool fibre proteomics. The proposed research complemented NZ existing wool quality traits programme and provided the opportunity to examine the effect of diet restriction on the protein composition of wool. This collaboration was successfully concluded with the finding of significant differences between the animals fed control and restricted diets (Almeida *et al.,* 2014). One of the other benefits of the COST researcher’s visit was a vision on research interests in the form of a seminar, awareness of some of his other publications and numerous informal meetings, in addition to the establishment of future collaborations and also some important consultations and discussions on proteomics equipment and software issues.

### Potentials and drawbacks

There is thus a current wave of interest in the application of proteomics to animal research and it is important that the momentum gained is not dissipated but is captured to yield the maximum benefit possible. How can this be achieved? The equipment needed to deliver technology for proteomics is likely to remain expensive for the foreseeable future, with few animal science institutes being willing to devote resources exclusively to proteomics; thus, it is important that centres of proteomic expertise remain open for collaboration with animal research. With the increasing recognition of the importance of research impact to the wider community, such laboratories should become more amenable to collaboration as direct links to public benefit and research impact can be established. Proteomics can identify disease biomarkers and vaccine candidates for economically important diseases of livestock and fish in aquaculture, as well as play a major role in species authentication to reassure consumers of the veracity of food products on supermarket shelves. Many of these applications have commercial application and it can be expected that veterinary and animal diagnostics, along with vaccine and animal health product companies, will recognize the need to explore the proteome of blood and tissues from experimental or natural disease investigations in order to determine the roles and potential actions of bioactive proteins and peptides.

A growing range of applications for proteomics of domestic animals, evidenced over the experience of the last few years, will extend the demand for expertise to apply the technology as access and the knowledge base increases. The ubiquitous use of 1D electrophoresis based on the Laemmli polyacrylamide gel technique throughout biological research indicates the need for protein analysis. As the use and value of proteomics with greater separation and more accurate protein identification within animal species increases, the potential applications of the method in animal science will multiply. As this occurs, it is likely that both equipment and reagent manufacturers become more familiar with the sector. For instance, a common problem in examination of the serum proteome is the overabundance of the proteins with the highest concentration, such as albumin and immunoglobulins. Kit-based methods are available to remove the 20 most abundant proteins in human serum, but as the method is antibody based it does not work in domestic species. Production of kits for the major farm animals would be of significant benefit to biomarker discovery in these species.

## Conclusion

It is clear that there is a need for proteomics to be included in future investigation of animal health, welfare and production. It is to be hoped that national and international funding bodies that allocate research funds will have the foresight to recognize that there is now a window of opportunity with technology. Expertise and motivation have been developed among a cohort of researchers to maximize the potential for future proteomic-based investigations. Among the active researchers who have demonstrated this potential, there is also a need to maintain the progress, especially after the COST Action on FAP is completed by November 2014. There is a need for the consortium to continue and to expand to include experts from beyond Europe either by formation of an Association or by linkage to an established group. Whatever happens there is no doubt that the use of proteomics in animal research has now gone beyond a few, isolated laboratories and can now be seen as a mainstream research tool of benefit across the spectrum of investigations into animal health, production and post-harvest processes. In particular, the natural continuation of our action will be realized through the presentation of the Horizon 2020 (H2020) research programme. One of the four pillars of H2020 is dedicated to food security, sustainable agriculture and forestry, marine and maritime and inland water research, in which proteomic investigation is perfectly integrated. Moreover, these activities are transversal so that the results and networking produced from the COST action will open novel lines of enquiry in the light of one health approach that links human to animal medicine, which is one of the hot topics within the H2020 themes.
